# Pseudoexfoliation Syndrome—Clinical Characteristics of Most Common Cause of Secondary Glaucoma

**DOI:** 10.3390/jcm12103580

**Published:** 2023-05-21

**Authors:** Martyna Tomczyk-Socha, Wojciech Tomczak, Weronika Winkler-Lach, Anna Turno-Kręcicka

**Affiliations:** 1 Department of Ophthalmology, Wroclaw Medical University, 50-367 Wrocław, Poland; 2 Lower Silesian Oncology Center, 53-413 Wroclaw, Poland

**Keywords:** pseudoexfoliation syndrome, pseudoefoliation glaucoma, environmental factors, clinical characteristics

## Abstract

Pseudoexfoliation syndrome (XFS) is a condition in which excess material builds up not only in the structures of the anterior chamber but also throughout the body. The frequency of the syndrome varies significantly (0.3–18%) depending on the region and the method of examination. Environmental risk factors for XFS include a large number of sunny days, proximity to the equator, dietary factors such as higher consumption of coffee and tea, long-term alcohol consumption, exposure to UV, and outdoor work. The pathognomonic sign of XFS is the presence of white material on the lens capsule and other anterior chamber components. In addition, a characteristic Sampaolesi line can be observed during gonioscopy. Systemic alterations indicative of XFS have been observed in the extracellular matrix of the eyelid skin, the heart, lungs, liver, kidneys, gallbladder, meninges, and endothelium of the blood vessels. XFS is the most common cause of secondary open-angle glaucoma, which is called pseudoexfoliative glaucoma and is more severe than primary open-angle glaucoma. It is plausible that a combination of environmental factors and genetic alterations promotes the onset of pseudoexfoliation syndrome, which requires additional research.

## 1. Introduction

Pseudoexfoliation syndrome (XFS) is an age-related disease in which abnormal material accumulates in the anterior segment of the eyeball. The origin of the pseudoexfoliation material (XFSM) is associated with a dysregulation in the elastin synthesis process and the formation of aggregates of abnormal elastic fibers, accompanied by a significant reduction in collagen fibers [1].

XFS is considered the most common of the identifiable causes of glaucoma. XFS is the cause of secondary open-angle glaucoma, the so-called pseudoexfoliative glaucoma (XFG) associated with a higher risk of blindness than primary open-angle glaucoma (POAG) [2,3]. PEX is estimated to be the most common cause of glaucoma-related blindness worldwide [4].

Due to the blockage of the trabecular meshwork and the significant fluctuation in intraocular pressures (IOP), there is a change in the architecture of the trabecular structure and severe damage to the optic nerve at the time of diagnosis. Those anatomical changes result in greater resistance to conservative treatment, and the prognosis in XFG is much worse than in POAG [5].

Various genetic and nongenetic factors (such as environmental factors) are known to be linked to the development of XFG. According to the literature, the latest theory that may hold the answer to XFS pathogenesis comprises four factors [6]: changes in miRNA expression [7], disordered autophagy, the potential involvement of mitochondrial mutations, and a compromised aqueous–blood barrier [8]. Additionally, genetic changes such as polymorphism of the lysyl oxidase-like 1 (LOXL1) enzyme should be taken into consideration.

Therefore, this review aims to synthesise the current literature on the characteristics of pseudoexfoliation syndrome as stratified by epidemiology, clinical symptoms, influence of environmental factors, systemic manifestations and the characteristic of secondary glaucoma.

## 2. Methods

We performed a literature search of the PubMed database, identifying articles associated with XFS using the following terms: exfoliation syndrome, environmental factors, clinical characteristics, clinical features, systemic manifestations, and secondary glaucoma. The selection of articles for the review was based on the following criteria: (i) exfoliation syndrome AND environmental factors (n = 71); (ii) exfoliation syndrome AND clinical characteristic (n = 110); (iii) exfoliation syndrome AND clinical features (n = 94); (iv) exfoliation syndrome AND systemic manifestations (n = 55); (v) exfoliation syndrome AND secondary glaucoma (n = 183). After searching and evaluating, all selected papers were independently examined by the two authors.

## 3. Epidemiology of Pseudoexfoliation Syndrome

XFS is a worldwide disease, and the prevalence of the condition is probably significantly underestimated. The incidence varies depending on the publication, the region in which the study was performed, and the way in which the study was conducted. Currently, there are no studies on the prevalence of XFS in the population. Most published studies indicate the prevalence of XFS in a given group, e.g., among patients with glaucoma or cataracts, which significantly distorts the epidemiological data. In publications on prevalence, data vary from 0.3% (Mongolia) to 18% (Sweden) [9]. Hirvela reported that the prevalence of exfoliation increased with age (*p* = 0.0001). Uni- or bilateral exfoliation was seen in 14% of the age group of 70–74 years, in 28% of the group of 75–79 years, 26% of the group of 80–84 years, and 30% of patients aged 85 years [10]. The relationship between XFS frequency and latitude was also discussed. The closer to equator, the lower the incidence of XFS [11]. In patients under the age of 50, XFS is uncommon, and its prevalence rises with advancing age. XFS is more common in women than in men [10].

## 4. Influence of Several Risk Factors in Pseudoexfoliation Syndrome

Environmental factors are investigated and described in POAG as risk factors for the disease. In XFS, they were not initially studied, but after the discovery of the LOXL1 gene [12] in 2007 and their different effects and frequencies depending on geographical location, an intensive search for possible causes of these variabilities was initiated.

Stein et al. investigated the influence of factors on the occurrence of XFS and XFG. After analysing the average annual rainfall and snowfall, the average annual number of sunny days, and the average temperature in winter and summer, they found that more sunny days were associated with a higher incidence of XFS [13]. One could conclude from this that there should be the largest number of patients with XFS around the equator. In contrast, epidemiological data show exactly the opposite principle—the further from the equator, the more frequent the occurrence of XFS [11].

Diet and eating habits were also taken into account, and it was determined that high consumption of caffeinated drinks significantly increased the incidence of XFS. The risk of developing XFS is increased by 66% in individuals who consume more than three cups of coffee per day. In addition, caffeine increases homocysteine levels, which can further affect the development of XFS [14].

The effect of smoking on the incidence of XFS was also studied. Tijani et al. found that XFS was diagnosed in more than 50% of smoking patients, suggesting that smoking may be another factor contributing to the development this syndrome (*p* = 0.01) [15]. On the other hand, Kim et al. analysed the influence of many factors on the occurrence of XFS, including the number of pack-years per occurrence of XFS, finding no significant difference [16]. They also determined the effect of hypertension on the occurrence of XFS, finding no difference (*p* = 1.0) [15].

**Figure 1:**
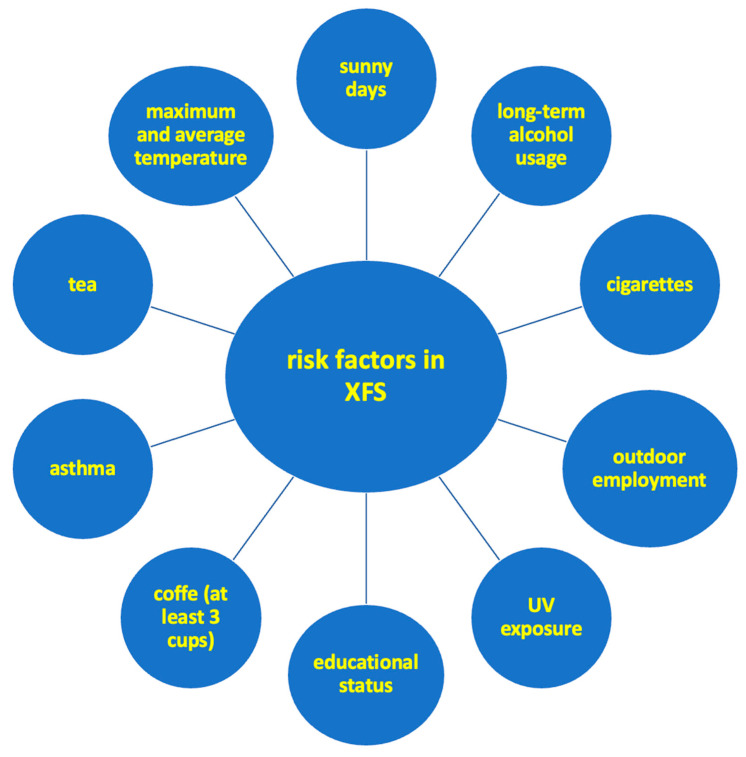
Influence of several risk factors in pseudoexfoliation syndrome.

According to the most recent research, long-term alcohol usage is related to an increased risk of XFS and XFG [17]. Ocular UV exposure throughout a lifetime and outdoor employment are strongly connected with the development of XFS [18]. Educational status (literacy was associated with a lower incidence of PEX), asthma, average lifespan, daily tea consumption, weighted maximum temperature, and weighted mean temperature were identified as risk factors among Pathans [19]. A summary of several risk factors that may be associated with PXS is shown in Figure 1.

## 5. The Clinical Characteristics of Pseudoexfoliation Syndrome

XFS is most often diagnosed at an advanced stage; the diagnosis is based on the disease symptoms. Therefore, in order to clearly identify XFS, clinical examination alone can be insufficient at the beginning stage of the disease and in pseudophakic patients.

White flaky dandruff-like deposits (consisting of abnormal fibrillo-granular protein) are visible in the anterior chamber. The pathognomonic symptom of XFS is the appearance of three separate surfaces on the lens capsule, which are visible when the pupil is dilated. In the centre, there is a whitish ring of relatively homogeneous structure, which corresponds to the pupil size before dilatation. Next, a white ring that is granular, frequently layered, and has uneven, jagged edges encircles the centre. These two annular zones are separated by a strip of clear lens surface without deposits. It is caused by the movements of the iris during the change in the width of the pupils when the posterior surface of the iris rubs against the anterior surface of the lens and thus wears away the material deposited on the lens capsule. The wiped-off XFSM is initially suspended in the aqueous humor. Then it deposits on other structures in the anterior eye segment, including the trabecular meshwork, blocking the drainage angle.

Iris alterations in the area of the pupillary sphincter muscle are another characteristic symptom. Iris translumination, also known as characteristic moth-eaten peripupillary iris appearance, is brought on by abrasion of the pigment epithelium of the iris caused by pupil movements. Deposits of XFSM on the pupillary margin of the iris are also quite common. 

The XFSM present on the lens capsule acts as sandpaper on the iris, contributing to the release of a large amount of pigment from the iris. The released pigment causes hyperpigmentation of the filtration angle as well as the formation of pigment deposits on the iris surface.

It is challenging to recognise XFS in the early stages of the disease. Still, the homogenous, dull appearance of the anterior lens capsule, resembling a clouded glass window, should be suspicious, especially if it is present in only one eye of the patient.

A little later, fine radial striations can be seen extending from the forming central disc. Examination in low light can help to identify these changes. Only when the layer of exfoliating material placed on the surface of the lens becomes thicker, the transparent zone between the central part and the peripheral ring begins to be visible. XFS can occur in both eyes, but asymmetry is very common.

There are currently no diagnostic procedures to confirm XFS. Only electron microscopy can reveal the characteristic material in the anterior segment of the eyeball [1].

Precipitates of pseudoexfoliative material and pigment might be seen on the corneal endothelium. Most often, they are diffuse and cover the central part of the endothelium. Sometimes the deposits may resemble a Krukenberg spindle [20].

The number of endothelial cells is reduced, but the thickness of the cornea in the centre is increased. All of these changes might lead to a faster disturbance of endothelial cell homeostasis. A slightly elevated IOP can cause corneal decompensation, opacity, and oedema. Patients with XFS also have a higher postoperative incidence of ocular oedema following cataract surgery [20].

On gonioscopy in patients with XFS, the characteristic Sampaolesi line can be observed; in other words, intense inhomogeneous pigmentation of the trabecular meshwork with a wavy band of pigment on the corneal endothelium anterior to the Schwalbe line is present [21]. The Sampaolesi line should be differentiated from the intense homogenous hyperpigmentation of the filtration angle present in the pigment dispersion syndrome. In some cases of XFS, the angle can be narrow or closed when the lens moves anteriorly due to zonular laxity.

The periocular changes in XFS include the development of eyelid laxity, conjunctival chalasis, tear film abnormalities, orbital fat atrophy after the administration of prostaglandin analogues, deficient orbital vascular supply and biomechanical changes in both the eyeball and the optic nerve [22].

PEX significantly affects the intraoperative and postoperative course of cataract removal. In PEX, due to zonular weakness, the ligament apparatus of the lens is damaged, which leads to a significantly increased risk of intraoperative complications, such as rupture of part of the zonules, rupture of the posterior capsule of the lens, transition of the vitreous body to the anterior segment of the eye, and drowning of the cataract masses in the vitreous chamber. In addition, the response of the iris to mydriasis drugs is incomplete, which significantly hinders and prolongs the course of the procedure.

In the postoperative course, mild chronic inflammation, displacement of the implanted lens, subluxation of the lens, opacification of the posterior lens capsule, and fibrosis and tightening of the lens capsule may occur.

**Figure 2:**
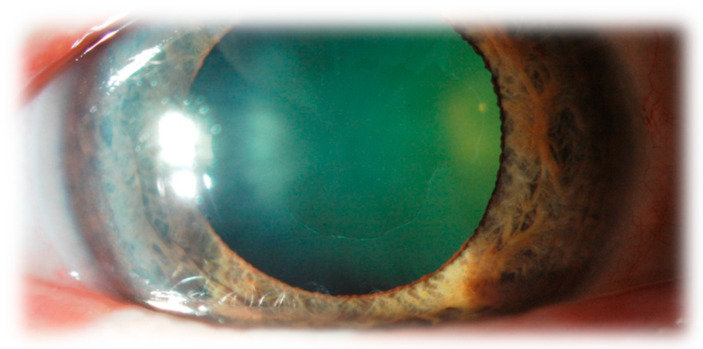
Characteristic dandruff-like deposits on the lens capsule.

It is also worth noting that the diagnosis of XFS in a patient who has undergone cataract extraction is significantly difficult due to the lack of a pathognomonic symptom that was present on the anterior surface of the lens capsule removed during the procedure. As a result, a significant number of people living with XFS remain undiagnosed. When there are no characteristic symptoms on the lens capsule, it is difficult to recognise XFS. Figure 2 shows characteristic dandruff-like deposits in the anterior segment.

## 6. Systemic Manifestations of Pseudoexfoliation Syndrome

In addition to visual problems, XFS can also lead to systemic disorders. Extracellular matrix in the tissues of the eye, eyelid skin, heart, lungs, liver, kidneys, gallbladder, meninges, and endothelium of blood vessels have been found to have alterations that are typical of XFS using an electron microscope [1,23].

Research suggests that XFS is often associated with disorders of the heart and blood vessels. For example, it has been demonstrated that patients with XFS have changes in blood flow both systemically and ocularly, as well as altered parasympathetic vascular control and baroreceptor sensitivity, increased vascular resistance, decreased blood flow velocity, endothelial dysfunction, and high levels of homocysteine [24].

XFS has been associated with cardiovascular diseases such as abdominal aortic aneurysm, transient ischemic attacks (TIA), angina pectoris, myocardial infarction and ischemic stroke [24]. 

A large database [25] was analysed for comorbidities that involve anomalies in elastin maintenance; chronic obstructive pulmonary disease, inguinal hernias, pelvic organ prolapse, obstructive sleep apnea, and atrial fibrillation were associated with XFS [24].

In addition to confirming an increased risk of respiratory, cardiovascular, and genitourinary disease, recent data have shown an increased risk of heart valve disease and benign prostatic hyperplasia in patients with XFS [26]. Common pathogenesis features of both atherosclerosis and XFS, such as oxidative stress and inflammation, may suggest that these grey–white deposits seen in the anterior segment and cardiovascular disorders are related or reflect different manifestations of the same process.

## 7. Pseudoexfoliation Syndrome as a Cause of Glaucoma

XFS is the most common identifiable cause of secondary glaucoma globally. It is estimated that up to half of the people with XFS will develop glaucoma in their lifetime. XFS patients have a 6 to 10 times higher risk of developing glaucoma compared to the healthy population [27]. In many cases, the high risk for IOP elevation and glaucoma development develops soon after XFS becomes clinically evident.

The pathomechanism of the development of glaucoma changes is associated with elevated IOP, which is secondary to increased resistance in the outflow of aqueous humour. XFS causes a mechanical blockage in the drainage angle by occluding the trabecular meshwork with XFSM and iris-released pigment. The accumulation of these substances in the trabecular meshwork results in degenerative changes and the remodelling of the Schlemm’s canal and adjacent structures.

The values of IOP are usually significantly greater in patients with XFG than those with POAG, and the daily pressure variations are far more significant (greater IOP difference between affected and contralateral unaffected eye). The daily distribution of IOP may be different from that of POAG. The highest values do not have to occur in the morning, as in other patients with glaucoma. Increases in the IOP can occur at any time of the day, which is why circadian IOP curves in the course of XFG are so important, especially in patients with a large progression of glaucomatous changes [28].

A positive relationship was observed between intraocular pressure and the degree of pigmentation of the drainage angle in XFS and possibly with the degree of trabecular dysfunction [29].

In addition, XFS leads to degenerative changes in blood vessels, chronic breakdown of the aqueous humour–blood barrier, and impaired blood flow in the eye’s blood vessels [28].

Each dilatation of the pupil causes the discharge of dispersed pigment from the iris, which, deposited in the filtration angle, can block it and lead to a significant increase in IOP. It is, therefore, essential to measure IOP in XFS patients again after pupil dilation.

When compared to POAG, the average age of diagnosis for XFG is significantly higher. In most cases, it does not manifest itself until beyond the age of 50. Patients diagnosed with XFS have shown no evidence of an enhanced male or female predisposition to the development of XFG. Nonetheless, it is expected that diagnosed men experience a more severe course of XFG than women [27].

The course of XFG, prognosis, and response to treatment are significantly worse than in POAG. Much more often, glaucoma progression cannot be stopped with pharmacological treatment, so more patients require glaucoma surgery.

The more frequent progression observed in XFG is due to higher mean IOP values, larger IOP spikes during the day than in POAG, older patient age, and greater visual field damage at diagnosis [28,29,30,31,32,33].

Konstas et al. [28] found that baseline IOP was higher in XFG patients than in POAG patients. Similar findings were reported by Tezel and Tezel [29], Lindblom and Thorburn [31], and Futa et al. [32]. In addition, Teus et al. [33] showed that among untreated patients, a higher level of IOP was associated with a greater loss of visual field (the MD, mean deviation coefficient, was examined).

It has not been determined yet whether the worse prognosis in XFG is associated with higher and more fluctuating IOP values during treatment, older age of patients, or a poorer reaction to treatment. However, the reported patients’ lower response to IOP-lowering drugs resulted in worsening IOP parameters and higher daily fluctuations, so the interplay of these factors makes a study difficult to conduct.

Blika and Saunte [34] demonstrated that only 8% of individuals with XFG had it under control with the use of a beta-blocker, in contrast to the 33% of patients who had POAG. In addition, Pohjanpelto [35] showed that in approximately ten years of follow-up, 35% of XFG patients required trabeculectomy to control IOP compared to 18% of POAG patients.

Konstas [36] investigated the relationship between the mean IOP in XFG and disease progression. He reported that progression occurred in:

- 28% of patients with an IOP of 17 mmHg or less,
- 43% of patients with an IOP of 18 to 19 mmHg,
- 70% of patients with an IOP of 20 mmHg or more.

The highest mean IOP value was 24.1 ± 5.4 mmHg in the group with stable glaucoma, and the lowest mean IOP in the group with progressive glaucoma was 29.2 ± 10.3 mmHg [36].

Some drugs have been found to work better in XFG than in POAG. The advantage of bimatoprost over latanoprost [37] in XFG and a better IOP-lowering effect of apraclonidine compared to POAG [38] have been demonstrated.

Very good results in IOP regulation can be obtained by performing laser trabeculoplasty, which increases and facilitates the outflow of aqueous humour through the trabecular meshwork. A greater reduction in IOP was obtained in XFG than in POAG. However, the effect of trabeculoplasty decreased over time, and the failure rate increased by 10% every 12 months [39].

## 8. Conclusions

XFS is a disease that causes a severe form of secondary open-angle glaucoma as well as systemic alterations. In spite of the fact that XFS can be found all over the world, its prevalence varies greatly from region to region. This suggests that the symptoms of XFS could be caused by either genetic or environmental causes.

Many studies are now being conducted to identify the precise origin of XFS, methods for diagnosing and treating XFS, and the systemic effects of XFS. As a result of the recent surge in interest in XFS, a great number of groundbreaking discoveries are currently being made public, notably in the realm of molecular genetics. We are learning more and more, but we still do not know the answer to the most important question, which is how XFSM is formed and whether we can cure a patient with XFS, preferably before glaucoma damage happens and the typical deposits of material show up. It is likely that a combination of environmental factors with genetic changes triggers the onset of pseudoexfoliation syndrome, which requires additional study to elucidate the cause of XFS and the therapeutic possibilities for this condition.

## Data Availability

No new data were created or analyzed in this study. Data sharing is not applicable to this article.

## References

[1] Schlötzer-Schrehardt U.M., Koca M.R., Naumann G.O., Volkholz H. (1992). Pseudoexfoliation syndrome. Ocular manifestation of a systemic disorder?. Arch. Ophthalmol..

[2] Schlotzer-Schrehardt U., Naumann G.O. (2006). Ocular and systemic pseudoexfoliation syndrome. Am. J. Ophthalmol..

[3] Ritch R., Schlotzer-Schrehardt U. (2001). Exfoliation syndrome. Surv. Ophthalmol..

[4] Liu Y., Allingham R.R. (2011). Molecular genetics in glaucoma. Exp. Eye Res..

[5] Ritch R., Schlotzer-Schrehardt U., Konstas A.G. (2003). Why is glaucoma associated with exfoliation syndrome?. Prog. Retin. Eye Res..

[6] Chakraborty M., Rao A. (2022). Alternate Causes for Pathogenesis of Exfoliation Glaucoma, a Multifactorial Elastotic Disorder: A Literature Review. Curr. Issues Mol. Biol..

[7] Tomczyk-Socha M., Tomczak W., Turno-Krecicka A. (2022). The Importance of MicroRNA Expression in Pseudoexfoliation Syndrome. Int. J. Mol. Sci..

[8] Kuchle M., Vinores S.A., Mahlow J., Green W.R. (1996). Blood-aqueous barrier in pseudoexfoliation syndrome: Evaluation by immunohistochemical staining of endogenous albumin. Graefes Arch. Clin. Exp. Ophthalmol..

[9] Ringvold A. (1999). Epidemiology of the pseudo-exfoliation syndrome. Acta Ophthalmol. Scand..

[10] Hirvelä H., Tuulonen A., Laatikainen L. (1995). Intraocular pressure and prevalence of glaucoma in elderly people in Finland: A population-based study. Int. Ophthalmol..

[11] Pasquale L.R., Jiwani A.Z., Zehavi-Dorin T., Majd A., Rhee D.J., Chen T., Turalba A., Shen L., Brauner S., Grosskreutz C. (2014). Solar exposure and residential geographic history in relation to exfoliation syndrome in the United States and Israel. JAMA Ophthalmol..

[12] Thorleifsson G., Magnusson K.P., Sulem P., Walters G.B., Gudbjartsson D.F., Stefansson H., Jonsson T., Jonasdottir A., Stefansdottir G., Masson G. (2007). Common sequence variants in the LOXL1 gene confer susceptibility to exfoliation glaucoma. Science.

[13] Stein J.D., Pasquale L.R., Talwar N., Kim D.S., Reed D.M., Nan B., Kang J.H., Wiggs J.L., Richards J.E. (2011). Geographic and climatic factors associated with exfoliation syndrome. Arch. Ophthalmol..

[14] Pasquale L.R., Wiggs J.L., Willett W.C., Kang J.H. (2012). The Relationship between caffeine and coffee consumption and exfoliation glaucoma or glaucoma suspect: A prospective study in two cohorts. Investig. Opthalmology Vis. Sci..

[15] Tijani M., Albaroudi N., Boutimzine N., Cherkaoui O., Laghmari M. (2017). Prevalence of exfoliation syndrome and cardiovascular diseases in patients scheduled for cataract surgery. J. Fr. Ophtalmol..

[16] Kim S., Lim S.H., Sung K.R., Yun S.C., Kim C.Y., Park K.H., Cha S.C. (2016). Prevalence of Pseudoexfoliation Syndrome and Associated Factors in South Koreans: The Korean National Health and Nutrition Examination Survey. Ophthalmic Epidemiol..

[17] Hanyuda A., Rosner B.A., Wiggs J.L., Negishi K., Pasquale L.R., Kang J.H. (2023). Long-term Alcohol Consumption and Risk of Exfoliation Glaucoma or Glaucoma Suspect Status among United States Health Professionals. Ophthalmology.

[18] Sureshkumar I., Gunalan V., Nareshkumar R.N., Sripriya K., Ronnie G., Sharada R., Asokan R. (2022). Evaluating the impact of ocular UV exposure for the development for pseudoexfoliation syndrome in a South Indian population. Clin. Exp. Optom..

[19] Arif S.A., Khan M.I., Nauman F., Arif M.A. (2021). The association between ethnicity, environmental and lifestyle factors and chronic disease in the development of pseudoexfoliation syndrome. Pak. J. Med. Sci..

[20] Ritch R. (2018). Ocular Findings in Exfoliation Syndrome. J. Glaucoma.

[21] Sampaolesi R., Zarate J., Croxato O. (1988). The chamber angle in exfoliation syndrome. Clinical and pathological findings. Acta Ophthalmol. Suppl..

[22] Detorakis E.T., Bontzos G., Drakonaki E.E., Spandidos D.A. (2021). Changes in peri-ocular anatomy and physiology in pseudoexfoliation syndrome (Review). Exp. Ther. Med..

[23] Streeten B.W., Li Z.Y., Wallace R.N., Eagle R.C., Keshgegian A.A. (1992). Pseudoexfoliative fibrillopathy in visceral organs of a patient with pseudoexfoliation syndrome. Arch. Ophthalmol..

[24] Mitchell P., Wang J.J., Smith W. (1997). Association of pseudoexfoliation syndrome with increased vascular risk. Am. J. Ophthalmol..

[25] Pompoco C.J., Curtin K., Taylor S., Paulson C., Shumway C., Conley M., Barker D.J., Swiston C., Stagg B., Ritch R. (2021). Summary of Utah Project on Exfoliation Syndrome (UPEXS): Using a large database to identify systemic comorbidities. BMJ Open Ophthalmol..

[26] Scharfenberg E., Rauscher F.G., Meier P., Hasenclever D. (2019). Pseudoexfoliation syndrome: Analysis of systemic comorbidities of 325 PEX-positive patients compared with 911 PEX-negative patients. Graefes Arch. Clin. Exp. Ophthalmol..

[27] Irkec M., Hollo G., Konstas A. (2015). Clinical Features of Exfoliative Glaucoma w Exfoliation Syndrome and Exfoliative Glaucoma.

[28] Konstas A.G., Mantziris D.A., Stewart W.C. (1997). Diurnal intraocular pressure in untreated exfoliation and primary open- angle glaucoma. Arch. Ophthalmol..

[29] Iwanejko M., Turno-Krecicka A., Tomczyk-Socha M., Kaczorowski K., Grzybowski A., Misiuk-Hojlo M. (2017). Evaluation of the anterior chamber angle in pseudoexfoliation syndrome. Adv. Clin. Exp. Med..

[30] Tezel G., Tezel T.H. (1993). The comparative analysis of optic disc damage in exfoliative glaucoma. Acta Ophthalmol..

[31] Lindblom B., Thorburn W. (1984). Functional damage at diagnosis of primary open angle glaucoma. Acta Ophthalmol..

[32] Futa R., Shimizu T., Furuyoshi N., Nishiyama M., Hagihara O. (1992). Clinical features of capsular glaucoma in comparison with primary open- angle glaucoma in Japan. Acta Ophthalmol..

[33] Teus M.A., Castejon M.A., Calvo M.A., Perez-Salaices P., Marcos A. (1998). Intraocular pressure as a risk factor for visual field loss in pseudoexfoliative and in primary open-angle glaucoma. Ophthalmology.

[34] Blika S., Saunte E. (1982). Timolol maleate in the treatment of glaucoma simplex and glaucoma capsulare. A three-year follow up study. Acta Ophthalmol..

[35] Pohjanpelto P. (1986). Influence of exfoliation syndrome on prognosis in ocular hypertension greater than or equal to 25 mm. A long-term follow-up. Acta Ophthalmol..

[36] Konstas A.G., Hollo G., Astakhov Y.S., Teus M.A., Akopov E.L., Jenkins J.N., Stewart W.C. (2004). Factors associated with long-term progression or stability in exfoliation glaucoma. Arch. Ophthalmol..

[37] Konstas A.G., Hollo G., Irkec M., Tsironi S., Durukan I., Goldenfeld M., Melamed S. (2007). Diurnal IOP control with bimatoprost versus latanoprost in exfoliative glaucoma: A crossover, observer-masked, three-centre study. Br. J. Ophthalmol..

[38] Konstas A.G., Maltezos A., Mantziris D.A., Sine C.S., Stewart W.C. (1999). The comparative ocular hypotensive effect of apraclonidine with timolol maleate in exfoliation versus primary open-angle glaucoma patients. Eye.

[39] Threlkeld A.B., Hertzmark E., Sturm R.T., Epstein D.L., Allingham R.R. (1996). Comparative study of the efficacy of argon laser trabeculoplasty for exfoliation and primary open-angle glaucoma. J. Glaucoma.

